# Supplementary material for: Acinetobacter baylyi regulates type IV pilus synthesis by employing two extension motors and a motor protein inhibitor
Source: Nat Commun. 2021 Jun 18;12:3744. doi: 10.1038/s41467-021-24124-6 (PMC8213720; doi:10.1038/s41467-021-24124-6)
Supplement: Supplementary file 1 — Supplementary Information [file 41467_2021_24124_MOESM1_ESM.pdf]

# Supplementary Information for:

# *Acinetobacter baylyi* regulates type IV pilus synthesis by employing two extension motors and a motor protein inhibitor

Courtney K. Ellison[1,2*], Triana N. Dalia[3], Catherine A. Klancher[3], Joshua W. Shaevitz[1*], Zemer Gitai[2*], and Ankur B. Dalia[3*]

1. Lewis-Sigler Institute for Integrative Genomics, Princeton University, Princeton, NJ
2. Department of Molecular Biology, Princeton University, Princeton, NJ
3. Department of Biology, Indiana University, Bloomington, IN

*Correspondence to: c.ellison@princeton.edu, shaevitz@princeton.edu, zgitai@princeton.edu, ankdalia@indiana.edu

**This file includes:**

Supplementary figures 1-8
Supplementary tables 1-4

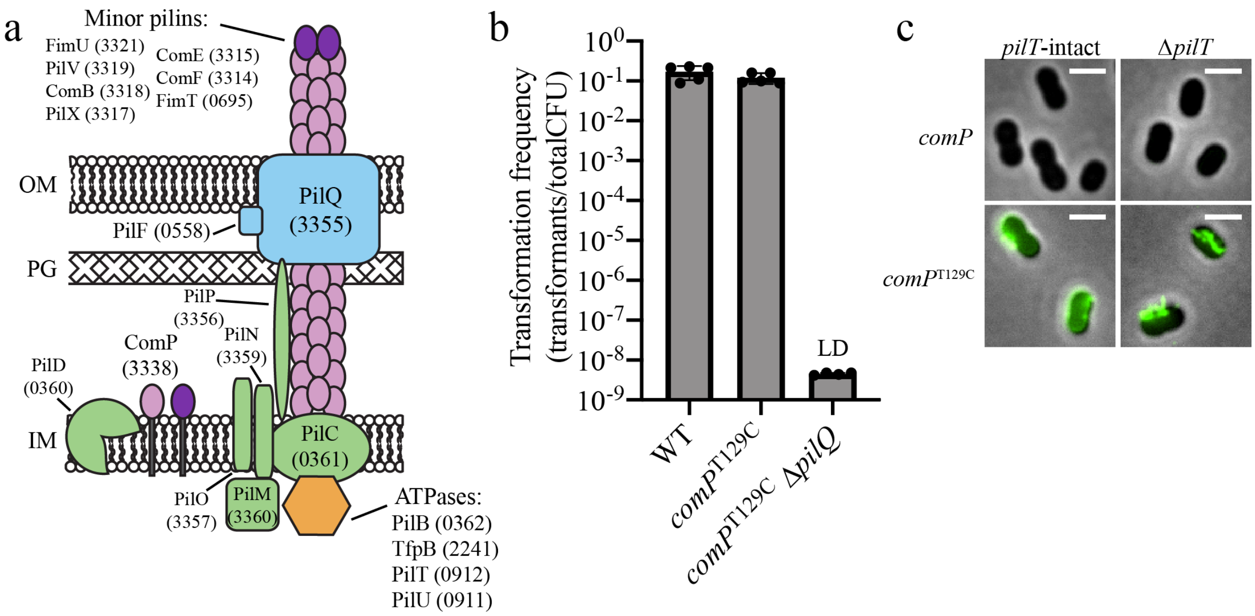


**Supplementary figure 1**. The strain for labeling T4P in *A. baylyi* is fully functional. (a) Schematic of assembled T4P components found in *A. baylyi*. Numbers in parentheses are ACIAD numbers associated with each component. OM, outer membrane; PG, peptidoglycan; IM, inner membrane. Inner membrane components are shown in green. Outer membrane components are shown in blue. Pilins are pink, and minor pilins are purple. Motor proteins are indicated by the gold hexagon. (b) Natural transformation assays of indicated strains. Each data point represents a biological replicate (WT n = 5; *comP*$^{T129C}$ n = 5; *comP*$^{T129C}$ Δ*pilQ* n = 4) and bar graphs indicate the mean ± SD. The transformation frequency of the Δ*pilQ* strain was below the limit of detection, indicated by LD. (c) Representative images of indicated strains labeled with AF488-mal with background fluorescence subtracted. Scale bars, 2 μm. Source data are provided as a Source Data file.

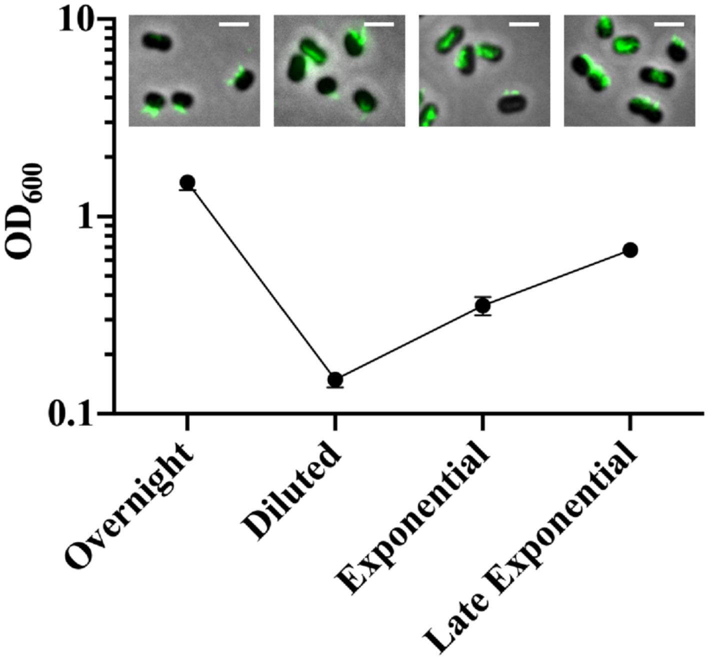


**Supplementary figure 2**. *A. baylyi* makes T4P throughout all growth phases. Representative images of the Δ*pilT* mutant labeled with AF488-mal at the indicated growth phase (n = 2). Error bars indicate the mean ± SD. Scale bars, 2 µm.

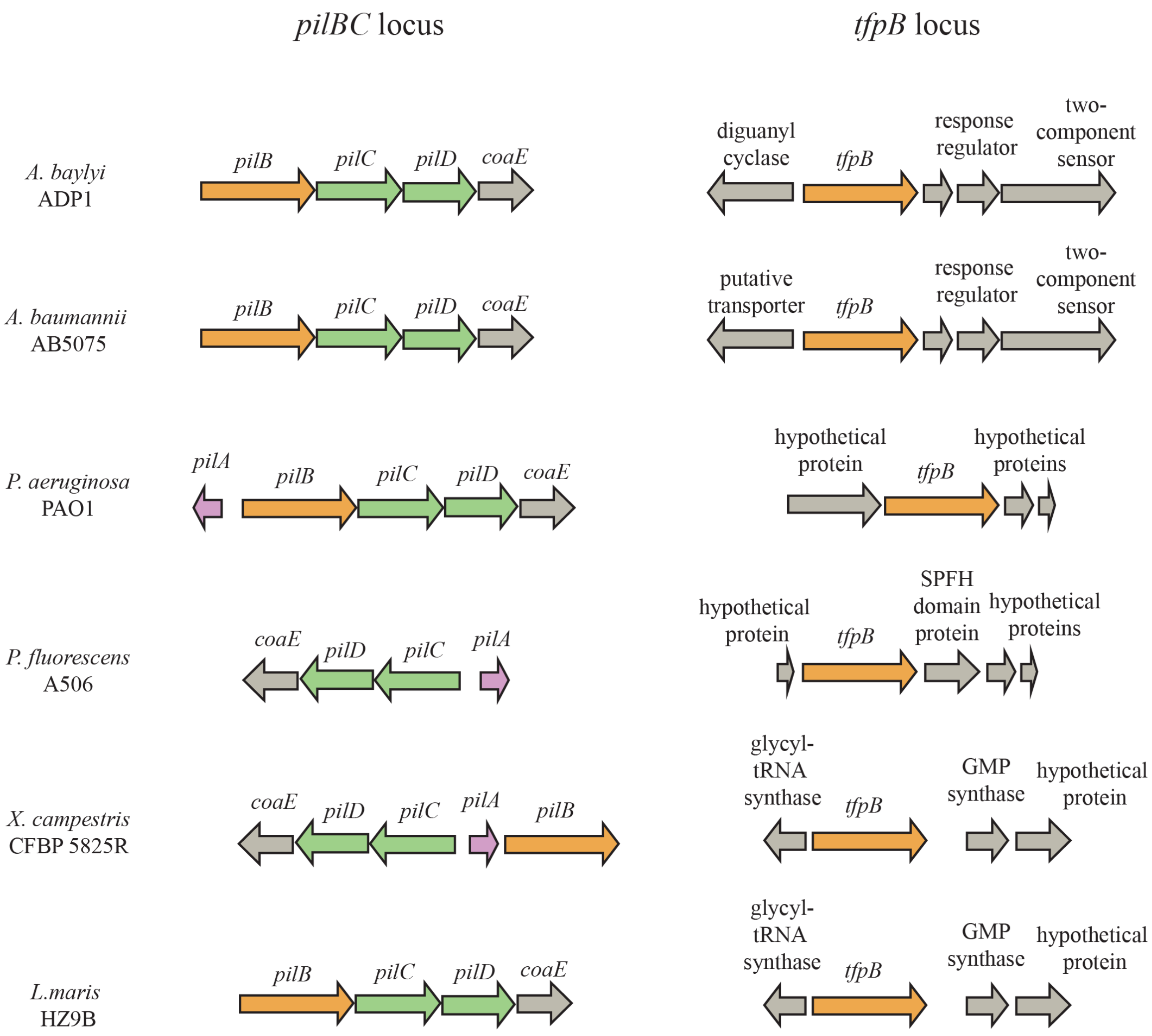


**Supplementary figure 3**. Schematic showing gene synteny of *pilBC* and *tfpB* loci in select species from the phylogenetic analysis shown in figure 2d. *P. fluorescens* lacks a PilB homologue and instead possesses only a *tfpB* gene. IMG accession numbers for *tfpB* and *pilB* genes are available in Supplementary table 2.

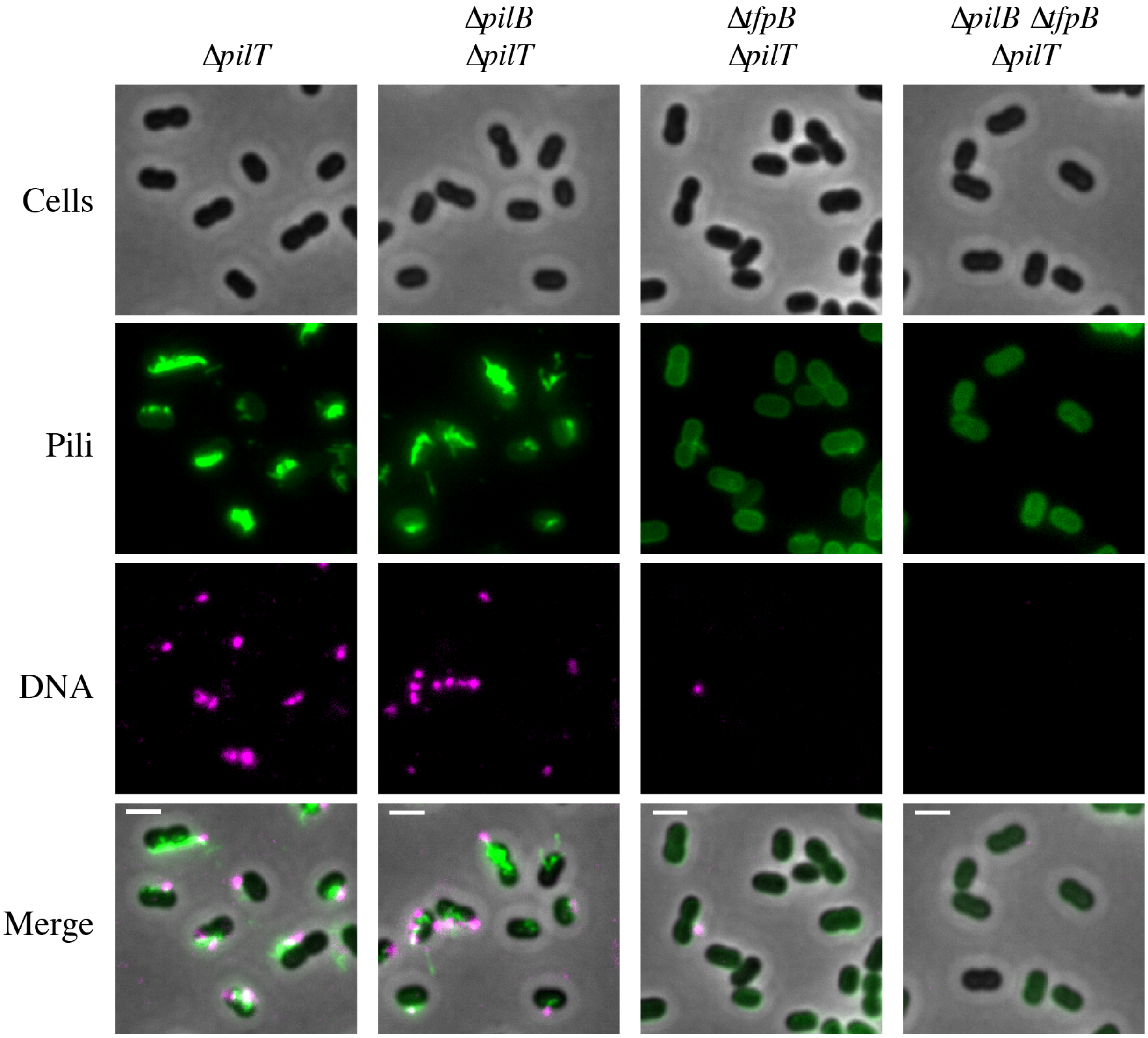


**Supplementary figure 4**. DNA binding in extension motor mutants. Representative images of cells with AF488-mal labeled pili incubated with fluorescently-labeled DNA. Scale bars, 2 μm.

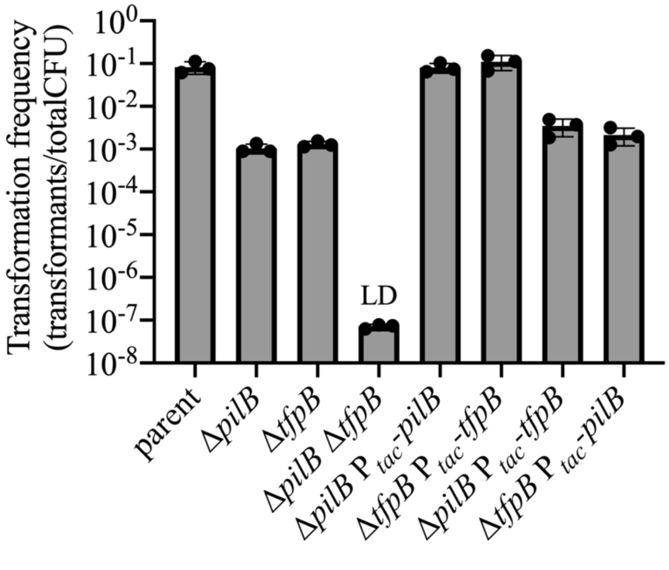


**Supplementary figure 5**. Natural transformation assays of the indicated strains. Each data point represents a biological replicate (n = 3) and bar graphs indicate the mean ± SD. The transformation frequency of the Δ*pilB* Δ*tfpB* strain was below the limit of detection, indicated by LD. Source data are provided as a Source Data file.

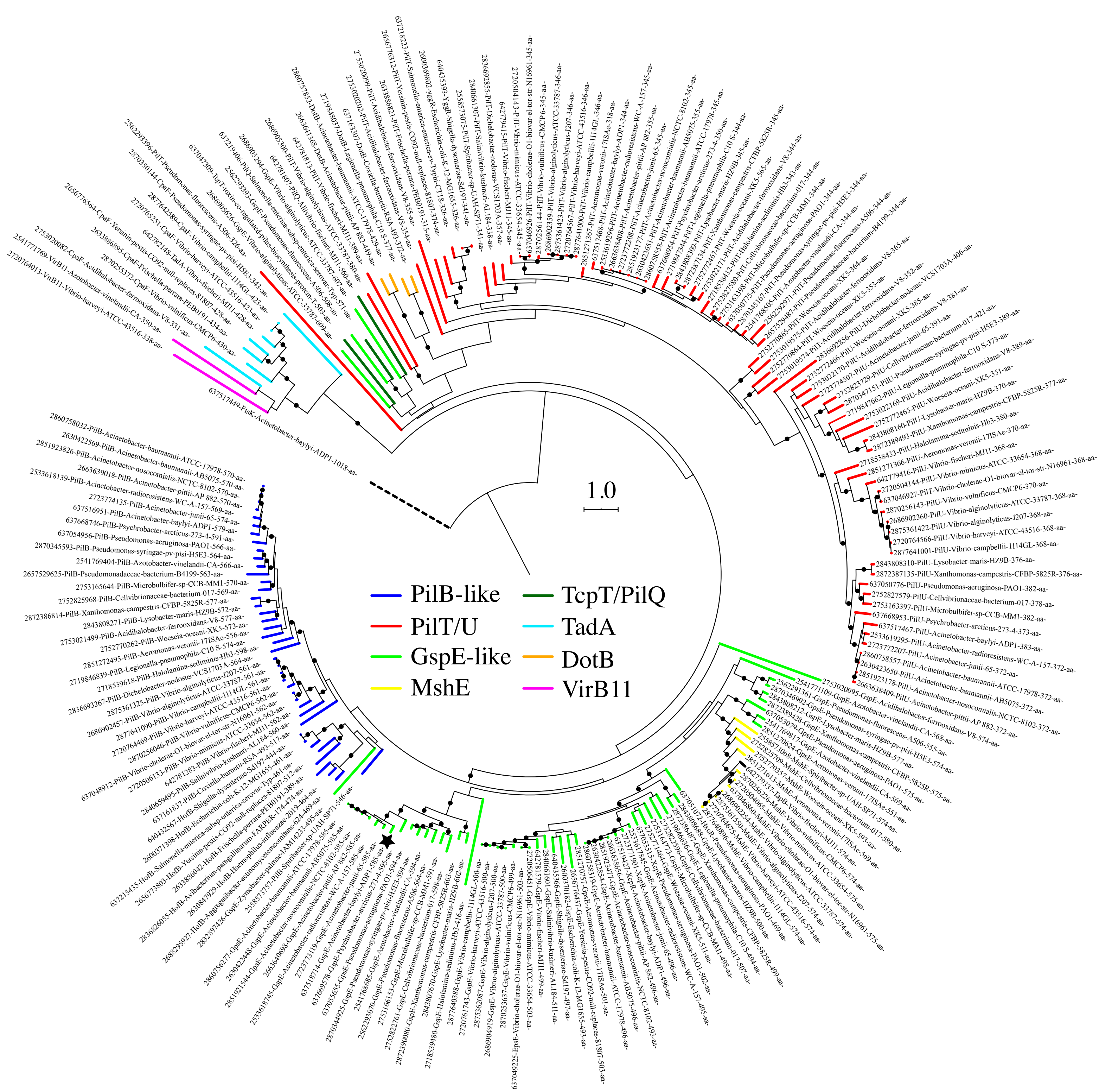


**Supplementary figure 6**. TfpB clusters with a group of other GspE-like proteins that are distinct from PilB proteins. A rooted phylogeny of TfpB homologues shown in Figure 2d with labels. PilQ in this context is not the secretin component, but is an alternate name for motors found in some species. Labels indicate IMG GeneID, annotated protein name, species, protein size. Nodes with bootstrap values greater than or equal to 70% are indicated by black circles. The black star denotes the tip of the branch representing *A. baylyi* TfpB.

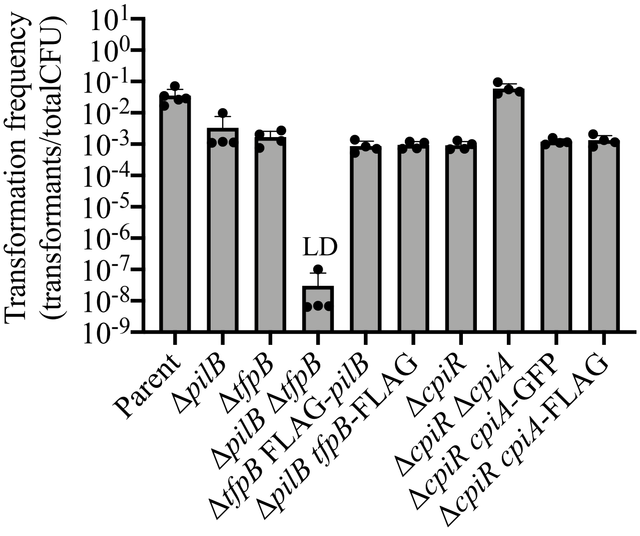


**Supplementary figure 7**. Natural transformation assays of indicated strains. Each data point represents a biological replicate (WT n = 5; all other strains n = 4) and bar graphs indicate the mean ± SD. The transformation frequency of the Δ*pilB* Δ*tfpB* strain was below the limit of detection, indicated by LD. Source data are provided as a Source Data file.

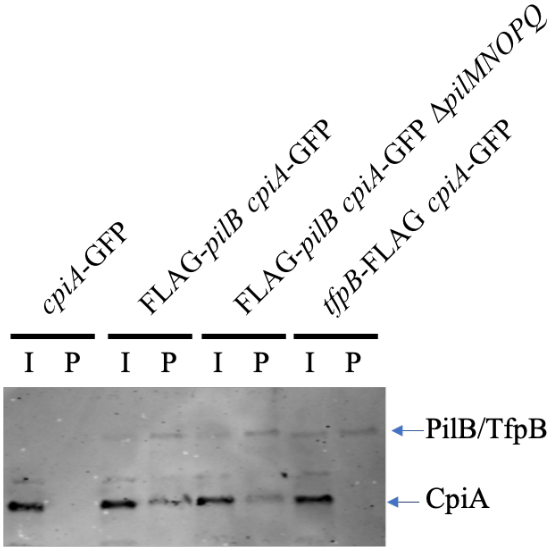


**Supplementary figure 8**. CpiA specifically interacts with PilB. Western blot showing coimmunoprecipitation experiments where FLAG-PilB or TfpB-FLAG were used as the bait proteins to test for interaction with CpiA-GFP as the prey protein in Δ*cpiR* mutant backgrounds. I, input sample; P, pulldown (coimmunoprecipitation) sample. Source data are provided as a Source Data file.

**Supplementary table 1**. Type IV pilus biosynthesis components in *A. baylyi* ADP1.

| **ACIAD#** | **Protein name** | **Predicted function** |
|---|---|---|
| ACIAD0360 | PilD | Prepilin peptidase |
| ACIAD0361 | PilB | Extension motor |
| ACIAD0362 | PilC | Inner membrane platform protein |
| ACIAD0558 | PilF | Pilotin |
| ACIAD0695 | FimT | Minor pilin |
| ACIAD0911 | PilU | Retraction motor |
| ACIAD0912 | PilT | Retraction motor |
| ACIAD2241 | TfpB | Extension Motor |
| ACIAD3314 | ComF | Minor pilin |
| ACIAD3315 | ComE | Minor pilin |
| ACIAD3317 | PilX | Minor pilin |
| ACIAD3318 | ComB | Minor pilin |
| ACIAD3319 | PilV | Minor pilin |
| ACIAD3321 | FimU | Minor pilin |
| ACIAD3338 | ComP | Major pilin |
| ACIAD3355 | PilQ | Outer membrane secretin |
| ACIAD3356 | PilP | Alignment complex protein |
| ACIAD3357 | PilO | Alignment complex protein |
| ACIAD3359 | PilN | Alignment complex protein |
| ACIAD3360 | PilM | Alignment complex protein |

**Supplementary table 2**. *tfpB* and *pilB* genes in species that harbor a TfpB homologue.

| **Species** | **TfpB IMG Gene ID** | **PilB IMG Gene ID** |
|---|---|---|
| *Acinetobacter baumannii* 17978 | 2860756277 | 2860758032 |
| *Acinetobacter baumannii* AB5075 | 2630422448 | 2630422569 |
| *Acinetobacter nosocomialis* NCTC 8102 | 2851921544 | 2851923826 |
| *Acinetobacter pittii* AP 882 | 2663640698 | 2663639018 |
| *Acinetobacter radioresistens* WC A 157 | 2533618745 | 2533618139 |
| *Acinetobacter junii* 65 | 2723773119 | 2723774135 |
| *Acinetobacter baylyi* ADP1 | 637518714 | 637516951 |
| *Psychrobacter arcticus* 273 | 637669578 | 637668746 |
| *Pseudomonas aeruginosa* PAO1 | 637055655 | 637054956 |
| *Pseudomonas syringae* pv pisi H5E3 | 2870344925 | 2870345593 |
| *Azobacter vinelandii* CA | 2541768685 | 2541769404 |
| *Pseudomonas fluorescens* A506 | 2562293070 | |
| *Microbulbifer* sp. CCB MM1 | 2753166153 | 2753165644 |
| *Cellvibrionaceae* bacterium 017 | 2752822761 | 2752825968 |
| *Xanthomonas campestris* CFBP 5825R | 2872390080 | 2872386814 |
| *Lysobacter maris* HZ9B | 2843807670 | 2843808271 |

**Supplementary table 3**. Bacterial strains used in this study.

| **CE#** | **ZG#** | **TND/ SAD#** | **Strain name in manuscript** | **Genotype** | **Figure ('S' denotes Supplementary figure)** |
|---|---|---|---|---|---|
| CE1 | | SAD631 | Wildtype (WT) | *Acinetobacter baylyi* ADP1 | S1B, S1C |
| CE100 | | SAD2537 | Pil-cys or Parent | ADP1 *comP*$^{T129C}$ | 1A, 1B, 1D, 2A, 3B, S1B, S1C, S5, S7 |
| CE329 | ZG1709 | | Δ*pilT* | ADP1 Δ*pilT* | S1C |
| CE317 | | TND0779 | Pil-cys Δ*pilT* or Parent | ADP1 *comP*$^{T129C}$ Δ*pilT*::*spec* | 1A, 1D, 2B, S1C, S2, S4 |
| | | TND0114 | Δ*comP* | ADP1 Δ*comP*::*kan* | 2A |
| CE150 | ZG1680 | | Δ*pilC* | ADP1 *comP*$^{T129C}$ Δ*pilC* | 1B, 1D |
| CE16 | ZG1678 | | Δ*pilY1* | ADP1 *comP*$^{T129C}$ Δ*pilY1* | 1B, 1D |
| CE66 | ZG1679 | | Δ*pilQ* | ADP1 *comP*$^{T129C}$ Δ*pilQ* | 1B, 1D, S1B |
| CE286 | | TND2544 | Δ*comEA* | ADP1 *comP*$^{T129C}$ Δ*comEA*::*kan* | 1B, 1D |
| CE287 | | TND2546 | Δ*comEC* | ADP1 *comP*$^{T129C}$ Δ*comEC*::*kan* | 1B, 1D |
| CE289 | | TND2556 | Δ*comM* | ADP1 *comP*$^{T129C}$ Δ*comM*::*kan* | 1B, 1D |
| CE22 | ZG1683 | | Δ*pilB* | ADP1 *comP*$^{T129C}$ Δ*pilB* | 2A, 2C, 4C, S5, S7 |
| CE67 | ZG1685 | | Δ*tfpB* | ADP1 *comP*$^{T129C}$ Δ*tfpB* | 2A, 2C, 4C, S5, S7 |
| CE54 | ZG1684 | | Δ*pilB* Δ*tfpB* | ADP1 *comP*$^{T129C}$ Δ*pilB* Δ*tfpB*::*kan*$^{R}$ | 2A, 2C, 4C, S5, S7 |
| CE310 | ZG1673 | | Δ*pilB* Δ*pilT* | ADP1 *comP*$^{T129C}$ Δ*pilB* Δ*pilT*::*spec*$^{R}$ | 2B, 2C, 4B, S4 |
| CE311 | ZG1674 | | Δ*tfpB* Δ*pilT* | ADP1 *comP*$^{T129C}$ Δ*tfpB* Δ*pilT*::*spec*$^{R}$ | 2B, 2C, 4B, S4 |
| CE316 | ZG1677 | | Δ*pilB* Δ*tfpB* Δ*pilT* | ADP1 *comP*$^{T129C}$ Δ*pilB* Δ*tfpB*::*kan*$^{R}$ Δ*pilT*::*spec*$^{R}$ | 2B, 2C, S4 |
| CE263 | | TND1201 | Δ*cpiR* | ADP1 *comP*$^{T129C}$ Δ*cpiR*::*kan*$^{R}$ | 3B, 4A, S7 |
| CE264 | | SAD2792 | Δ*cpiR* Δ*cpiA* | ADP1 *comP*$^{T129C}$ Δ*cpiRA*::*kan* | 3B, 4A, S7 |
| | | TND2260 | $P_{tac}$-*cpiA* | ADP1 *comP*$^{T129C}$ igACIAD0096-ACIAD0098::*Cm*$^{R}$-$P_{tac}$-*cpiA* | 3B |
| | | TND2093 | $P_{cpiA}$-GFP Parent | *V. cholerae* E7946 *Sm*$^{R}$ Δ*lacZ*::*spec*$^{R}$-$P_{cpiA}$-*gfp* | 3C |
| | | TND2126 | $P_{cpiA}$-GFP $P_{BAD}$-*cpiR* | *V. cholerae* E7946 *Sm*$^{R}$ Δ*lacZ*::*spec*$^{R}$-$P_{cpiA}$-*gfp* ΔVCA0692::*Carb*$^{R}$-$P_{BAD}$-*cpiR* | 3C |
| | | TND1335 | *cpiA*-FLAG | ADP1 *comP*$^{T129C}$ *cpiA*-3X FLAG | 3D |
| | | TND1284 | *cpiA*-FLAG Δ*cpiR* | ADP1 *comP*$^{T129C}$ *cpiA*-3X FLAG Δ*cpiR*::*kan*$^{R}$ | 3D, S7 |
| CE282 | | SAD2808 | Δ*pilB* Δ*cpiR* | ADP1 *comP*$^{T129C}$ Δ*pilB* Δ*cpiR*::*kan*$^{R}$ | 4A, 4C |
| CE283 | | SAD2806 | Δ*tfpB* Δ*cpiR* | ADP1 *comP*$^{T129C}$ Δ*tfpB* Δ*cpiR*::*kan*$^{R}$ | 4A, 4C |
| CE284 | | SAD2809 | Δ*pilB* Δ*cpiR* Δ*cpiA* | ADP1 *comP*$^{T129C}$ Δ*pilB* Δ*cpiRA*::*kan*$^{R}$ | 4A |
| CE285 | | SAD2807 | Δ*tfpB* Δ*cpiR* Δ*cpiA* | ADP1 *comP*$^{T129C}$ Δ*tfpB* Δ*cpiRA*::*kan*$^{R}$ | 4A, 4C |
| CE265 | | SAD2645 | Δ*cpiR* Δ*pilT* | ADP1 *comP*$^{T129C}$ Δ*cpiR*::*kan* Δ*pilT*::*spec*$^{R}$ | 4A, 4B |

| | | | | | |
|---|---|---|---|---|---|
| CE266 | | SAD2810 | Δ*cpiR* Δ*cpiA* Δ*pilT* | ADP1 *comP*$^{T129C}$ Δ*cpiRA*::*kan* Δ*pilT*::*spec*$^{R}$ | 4A |
| CE302 | | SAD2822 | Δ*pilB* Δ*cpiR* Δ*pilT* | ADP1 *comP*$^{T129C}$ Δ*pilB* Δ*cpiR*::*kan*$^{R}$ Δ*pilT*::*spec*$^{R}$ | 4A, 4B |
| CE303 | | SAD2820 | Δ*tfpB* Δ*cpiR* Δ*pilT* | ADP1 *comP*$^{T129C}$ Δ*tfpB* Δ*cpiR*::*kan*$^{R}$ Δ*pilT*::*spec*$^{R}$ | 4A, 4B |
| CE304 | | SAD2823 | Δ*pilB* Δ*cpiR* Δ*cpiA* Δ*pilT* | ADP1 *comP*$^{T129C}$ Δ*pilB* Δ*cpiRA*::*kan*$^{R}$ Δ*pilT*::*spec*$^{R}$ | 4A |
| CE305 | | SAD2821 | Δ*tfpB* Δ*cpiR* Δ*cpiA* Δ*pilT* | ADP1 *comP*$^{T129C}$ Δ*tfpB* Δ*cpiRA*::*kan*$^{R}$ Δ*pilT*::*spec*$^{R}$ | 4A, 4B |
| | | TND2904 | Δ*tfpB* FLAG-*pilB* | ADP1 *comP*$^{T129C}$ Δ*tfpB::kan*$^{R}$ 3X FLAG-*pilB* | S7 |
| | | TND2892 | Δ*pilB tfpB*-FLAG | ADP1 *comP*$^{T129C}$ Δ*pilB::kan*$^{R}$ *tfpB*-3X FLAG | S7 |
| | | TND1296 | *cpiA*-GFP Δ*cpiR* | ADP1 *comP*$^{T129C}$ *cpiA*-GFP Δ*cpiR::kan*$^{R}$ | S7, S8 |
| | | TND2337 | FLAG-*pilB* | ADP1 *comP*$^{T129C}$ 3X FLAG-*pilB* | 3D |
| CE608 | ZG1784 | | FLAG-*pilB* Δ*cpiR* | ADP1 *comP*$^{T129C}$ 3X FLAG-*pilB* Δ*cpiR::kan*$^{R}$ | 3D, 4D |
| | | TND2383 | FLAG-*pilB cpiA*-GFP Δ*cpiR* | ADP1 *comP*$^{T129C}$ 3X FLAG-*pilB cpiA*-GFP Δ*cpiR::kan*$^{R}$ | 4D, S8 |
| CE626 | ZG1785 | | FLAG-*pilB cpiA*-GFP Δ*cpiR* Δ*pilMNOPQ* | ADP1 *comP*$^{T129C}$ 3X FLAG-*pilB cpiA*-GFP Δ*cpiR::kan*$^{R}$ Δ*pilMNOPQ::zeo*$^{R}$ | S8 |
| CE656 | ZG1786 | | FLAG-*tfpB cpiA*-GFP Δ*cpiR* | ADP1 *comP*$^{T129C}$ *tfpB*-3X FLAG *cpiA*-GFP Δ*cpiR::kan*$^{R}$ | S8 |
| CE539 | ZG1789 | | Δ*pilB* $P_{tac}$-*pilB* | ADP1 *comP*$^{T129C}$ Δ*pilB* Δ*vanAB*::*kan*$^{R}$-$P_{tac}$-*pilB* | S5 |
| CE540 | ZG1790 | | Δ*tfpB* $P_{tac}$-*tfpB* | ADP1 *comP*$^{T129C}$ Δ*tfpB* Δ*vanAB*::*kan*$^{R}$-$P_{tac}$-*tfpB* | S5 |
| CE772 | ZG1791 | | Δ*pilB* $P_{tac}$-*tfpB* | ADP1 *comP*$^{T129C}$ Δ*pilB* Δ*vanAB*::*kan*$^{R}$-$P_{tac}$-*tfpB* | S5 |
| CE773 | ZG1792 | | Δ*tfpB* $P_{tac}$-*pilB* | ADP1 *comP*$^{T129C}$ Δ*tfpB* Δ*vanAB*::*kan*$^{R}$-$P_{tac}$-*pilB* | S5 |

CE strains available upon request from CKE, ZG strains available upon request from ZG, TND or SAD strains available upon request from ABD.

**Supplementary table 4**. Primers used for strain construction.

| **Primer name** | **Primer sequence 5' → 3' (overlapping regions underlined, point mutations in bold)** | **Description** |
|---|---|---|
| DOG0175 | AAACTATCCAGATAAGGGAAAGC | comP F1 |
| CEdalia87 | GAAGTTGTCGTACATTTCCA**ACA**TACAGCGCCACTAGCATATG | comPT129C R1 |
| CEdalia88 | CATATGCTAGTGGCGCTGTA**TGT**TGGAAATGTACGACAACTTC | comPT129C F2 |
| DOG0178 | AGGATCTGTAATGACGGGTTGAG | comP R2 |
| DOG0176 | GTCGACGGATCCCCGGAATCATAAAATTTCTCCACCAATGTTG | ΔcomP R1 |
| DOG0177 | GAAGCAGCTCCAGCCTACATGATAGTAGTACTATATGGCTTTAAAAG | ΔcomP F2 |
| ABD123 | ATTCCGGGGATCCGTCGAC | AbR cassette F |
| ABD124 | TGTAGGCTGGAGCTGCTTC | AbR cassette R |
| BBC1934 | GTTACAAAGTCAGGGACGTAAAG | ΔpilT F1 |
| BBC1935 | GTCGACGGATCCCCGGAATATCCATATTTCCCCGAAGATCG | ΔpilT R1 |
| BBC1936 | GAAGCAGCTCCAGCCTACATAAGAATAACGCTACTCGATCTG | ΔpilT F2 |
| BBC1939 | GGTATTCAGATTGATCGTCAGTTAG | ΔpilT R2 |
| CE389 | TCAGGAAGTTGCCTGAACCTGA | ΔpilC F1 |
| CE390 | ACAGAGCCCATTTGGAAAATTGGTGGCATTACTTGCGTTTTTTTTGCT | ΔpilC R1 |
| CE391 | AGCAAAAAAAACGCAAGTAATGCCACCAATTTTCCAAATGGGCTCTGT | ΔpilC F2 |
| CE392 | TTGAAAAGCTGGCTGGCCTTC | ΔpilC R2 |
| CE136 | TTGGTCGATAATGCAGATCAGCT | ΔpilY1 F1 |
| CE137 | GGGGTGCTTTGCTCATACCATTTTAAGAGATACTGAGTGTATATCGATTTTTTCAT | ΔpilY1 R1 |
| CE138 | ATGAAAAAATCGATATACACTCAGTATCTCTTAAAATGGTATGAGCAAAGCACCCC | ΔpilY1 F2 |
| CE139 | GCAGGGATATCAGATTTTAACTGTGC | ΔpilY1 R2 |
| CE85 | AATAAGAGCGAATAGCAAACTAATTGTCG | ΔpilQ F1 |
| CE340 | TTTCCAACAATAGTGTCATTAACTATTCGAGTCGTAAAAACGTTAAAATCTGTTCTCAT | ΔpilQ R1 (clean deletion) |
| CE341 | ATGAGAACAGATTTTAACGTTTTTACGACTCGAATAGTTAATGACACTATTGTTGGAAA | ΔpilQ F2 (clean deletion) |
| CE88 | AGCGTATAACGATCAATTACTTCGC | ΔpilQ R2 |
| BBC1891 | AATCACTTGAAGTGCAGATTCG | ΔcomEA F1 |
| BBC3451 | GTCGACGGATCCCCGGAATATGACGTATTGACATGAATTAACC | ΔcomEA R1 |
| BBC3452 | GAAGCAGCTCCAGCCTACATAAGAGGCTGAGTCATGCTCAAAATAC | ΔcomEA F2 |
| BBC1894 | ATCTTCACCTTCTCCAAACTGG | ΔcomEA R2 |
| DOG0180 | AGTATTTGAGTTCGGGTATTATTGC | ΔcomEC F1 |
| DOG0181 | GTCGACGGATCCCCGGAATCATGGCAATGCCAGCAATC | ΔcomEC R1 |
| DOG0182 | GAAGCAGCTCCAGCCTACAAAGGGAGCGATTCATTTTAAGTTTG | ΔcomEC F2 |
| DOG0183 | TTACTCAAGCCGTTACAGTTCTG | ΔcomEC R2 |
| DOG0140 | GTTGCTGCATTTGTTCGATCTG | ΔcomM F1 |
| DOG0141 | GTCGACGGATCCCCGGAATCATACTATTATTGTTCCATTATGGTGC | ΔcomM R1 |
| DOG0142 | GAAGCAGCTCCAGCCTACATATCGCAGTGAACATAGCTAAAA | ΔcomM F2 |
| DOG0143 | ATCAGTGGTTGGGAAGGTG | ΔcomM R2 |

| | | |
|---|---|---|
| CE23 | TTATGACTATCAATCTCAAGCACATGCT | ΔpilB F1 or 3XFLAG-pilB F1 |
| CE93 | ATTCACTGGTTACACGATTAATTTCCTGAAACTTAGGTGGTGTTGTAAATGCTG | ΔpilB R1 |
| CE94 | CAGCATTTACAACACCACCTAAGTTTCAGGAAATTAATCGTGTAACCAGTGAAT | ΔpilB F2 |
| CE26 | CTTTCATGGCGTGTTTGTCGTG | ΔpilB R2 or 3XFLAG-pilB R2 |
| CE210 | AAGCCAACCATAATCCGATTGTGAC | ΔtfpB F1 or tfpB-3XFLAG F1 |
| CE328 | TGGGACCACCCTTAAGACTTCTTCCCATTGTGTATCTATTTCAAAATGATATGTCAT | ΔtfpB R1 (in frame) |
| CE329 | ATGACATATCATTTTGAAATAGATACACAATGGGAAGAAGTCTTAAGGGTGGTCCCA | ΔtfpB F2 (in frame) |
| CE213 | CTCACGCCACCCAATCAAGAC | ΔtfpB R2 or tfpB-3XFLAG R2 |
| CE240 | GTCGACGGATCCCCGGAATTTATTTCTCCCCCACACCATTCAC | ΔtfpB R1 (AbR disruption) |
| CE241 | GAAGCAGCTCCAGCCTACATAAAAGGTTAGTTTAGATCTTAAGATTCACCTCAT | ΔtfpB F2 (AbR disruption) |
| DOG0130 | CCTCTCCACTCGTTTCTAAAGAAC | ΔcpiR or ΔcpiA or cpiA-3X FLAG F1 or cpiA-GFP F1 |
| DOG0131 | GTCGACGGATCCCCGGAATCATAAGCCGATACTATCTGGATGG | ΔcpiR R1 |
| DOG0132 | GAAGCAGCTCCAGCCTACAGAAGTTCAATGTCAGTTAAAACATC | ΔcpiR F2 |
| DOG0133 | GACGTTGATTACGTAAATTTAATTCG | ΔcpiR or ΔcpiA or cpiA-3X FLAG R2 or cpiA-GFP R2 |
| BBC921 | GTCGACGGATCCCCGGAATTTGATCACTTACTAATTTCATTATCATTTAAATG | ΔcpiA R1 |
| BBC922 | GAAGCAGCTCCAGCCTACATAAAAAAGGACTGATTTCTCAGCCC | ΔcpiA F2 |
| BBC2565 | TCCACCACTTCCACCTGCATTTTCATGAAACACTTGCTGC | cpiA-3X FLAG R1 |
| BBC2570 | GCAGGTGGAGCAGGTGGATAAAAAAGGACTGATTTCTCAGCCC | cpiA-3X FLAG F2 |
| BBC2272 | GCAGGTGGAAGTGGTGGAGATTATAAAGACCATGATGGTGACTACAAGGATCACGACATTGATTATAAGGATGACGATGACAAAGCAGGTGGAGCAGGTGGA | 3X FLAG MIDDLE F |
| BBC2273 | TCCACCTGCTCCACCTGCTTTGTCATCGTCATCCTTATAATCAATGTCGTGATCCTTGTAGTCACCATCATGGTCTTTATAATCTCCACCACTTCCACCTGC | 3X FLAG MIDDLE R |
| DOG0229 | CAATTTCACACAGGATCCCGGGAGGAGGTAACGTAATGATAATGAAATTAGTAAGTGATCAAATTC | Amplify *cpiA* for $P_{tac}$-*cpiA* construct F |
| DOG0230 | TGTAGGCTGGAGCTGCTTCTTAATTTTCATGAAACACTTGCTGC | Amplify *cpiA* for $P_{tac}$-*cpiA* construct R |
| BBC3072 | CACCATTATGATGGCAATCGTATGATTCGAAGTATGTGAAAAATCTGC | Amplify $P_{cpiA}$ for $P_{cpiA}$-GFP F |
| BBC3074 | CCCGGGATCCTGTGTGAAATTGACTAGCAGTATTGTATTACAAAACTTTTG | Amplify $P_{cpiA}$ for $P_{cpiA}$-GFP R |
| BBC3075 | CAATTTCACACAGGATCCCGGGAGGAGGTCGGCTTATGGATATAGGTCGC | Amplify *cpiR* for $P_{BAD}$-*cpiR* construct F |
| BBC2560 | TGTAGGCTGGAGCTGCTTCTTACTTTTTAGTGTCTATCAAAGTAAAAATGAGATG | Amplify *cpiR* for $P_{BAD}$-*cpiR* construct R |
| CE383 | GAAAAATTTGTTGAGCAGTTGAATGATGG | pilMF1 |

| | | |
|---|---|---|
| CE1507 | GTCGACGGATCCCCGGAATCTTCTTTGGCCTACGATATAACCTG | ΔpilMR1 (AbR disruption) |
| CE1508 | GAAGCAGCTCCAGCCTACACGAATAGTTAATGACACTATTGTTGGAAA | ΔpilQF2 (AbR disruption) |
| BBC2565 | TCCACCACTTCCACCTGCATTTTCATGAAACACTTGCTGC | cpiA-GFP R1 |
| BBC2570 | GCAGGTGGAGCAGGTGGATAAAAAAGGACTGATTTCTCAGCCC | cpiA-GFP F2 |
| BBC279 | GCAGGTGGAAGTGGTGGACGTAAAGGAGAAGAACTTTTC | Amplify GFP for fusions F |
| BBC350 | TCCACCTGCTCCACCTGCGTTGTATAGTTCATCCATGCC | Amplify GFP for fusions R |
| BBC3303 | TCCACCACTTCCACCTGCCATTAGACCGCCTTGTGATTCTG | 3XFLAG-pilB R1 |
| BBC3304 | GCAGGTGGAGCAGGTGGATCAGCATTTACAACACCACC | 3XFLAG-pilB F2 |
| BBC3751 | TCCACCACTTCCACCTGCAGTTAATGGGACCACCCTTAAGAC | tfpB-3XFLAG R1 |
| BBC3752 | GCAGGTGGAGCAGGTGGATAGTAAAAGGTTAGTTTAGATCTTAAGATTCACC | tfpB-3XFLAG F2 |
| CE317 | GCAAACCACAAACATAATGTTTGAAATCC | vanAB F1 for ectopic expression constructs |
| CE264 | GTCGACGGATCCCCGGAATGTGTGACGACTCCTTATCACTTATTGG | vanAB R1 for vanAB::kanR replacement |
| CE265 | GAAGCAGCTCCAGCCTACAAAGTTCAGTTTTTCTCCTATACATTAAGCC | vanAB F2 for vanAB::kanR replacement |
| CE260 | TTATGATGTCGGGCGGCCGCTTCGGAATAGGAACTTCAAGATCCCC | R1 for vanAB ectopic expression constructs |
| CE406 | GAAGCAGCTCCAGCCTACA | F2 for vanAB ectopic expression constructs |
| CE176 | CCAAGACTATAAATAATCGACATGATCAATTTTAA | vanAB R2 for ectopic expression constructs |
| CE261 | CTTGAAGTTCCTATTCCGAAGCGGCCGCCCGACATCATAAC | Ptac promoter F |
| CE336 | GCTTAATTACCTCCTAATTGAATTCCTAGGC | Ptac promoter R |
| CE1205 | CAATTAGGAGGTAATTAAGCATGTCAGCATTTACAACACCACCTAAG | Ptac-pilBF for ectopic expression at vanAB locus |
| CE1206 | TGTAGGCTGGAGCTGCTTCTTATTCACTGGTTACACGATTAATTTCCTGTAACG | Ptac-pilBR for ectopic expression at vanAB locus |
| CE1207 | CAATTAGGAGGTAATTAAGCATGACATATCATTTTGAAATAGATACACAATGGTGTTT | Ptac-tfpBF for ectopic expression at vanAB locus |
| CE1208 | TGTAGGCTGGAGCTGCTTCCTAAGTTAATGGGACCACCCTTAAGACTT | Ptac-tfpBR for ectopic expression at vanAB locus |
