# Supplementary material for: Acinetobacter baylyi regulates type IV pilus synthesis by employing two extension motors and a motor protein inhibitor
Source: Nat Commun. 2021 Jun 18;12:3744. doi: 10.1038/s41467-021-24124-6 (PMC8213720; doi:10.1038/s41467-021-24124-6)
Supplement: Supplementary file 3 — Description of Additional Supplementary Files [file 41467_2021_24124_MOESM3_ESM.pdf]

Description of Additional Supplementary Files

File Name: Supplementary Data 1
Description: Relative abundance of Tn insertions in each gene for all of the Tn-seq datasets analyzed
