# Supplementary material for: Acinetobacter baylyi regulates type IV pilus synthesis by employing two extension motors and a motor protein inhibitor
Source: Nat Commun. 2021 Jun 18;12:3744. doi: 10.1038/s41467-021-24124-6 (PMC8213720; doi:10.1038/s41467-021-24124-6)
Supplement: Supplementary file 5 — Reporting Summary [file 41467_2021_24124_MOESM5_ESM.pdf]

nature research

Corresponding author(s): Courtney Ellison, Josh Shaevitz, Zemer Gitai, Ankur Dalia

Last updated by author(s): May 25, 2021

Reporting Summary

Nature Research wishes to improve the reproducibility of the work that we publish. This form provides structure for consistency and transparency in reporting. For further information on Nature Research policies, see our Editorial Policies and the Editorial Policy Checklist.

Statistics

For all statistical analyses, confirm that the following items are present in the figure legend, table legend, main text, or Methods section.

n/a	Confirmed	
☐	☒	The exact sample size (*n*) for each experimental group/condition, given as a discrete number and unit of measurement
☐	☒	A statement on whether measurements were taken from distinct samples or whether the same sample was measured repeatedly
☒	☐	The statistical test(s) used AND whether they are one- or two-sided<br>*Only common tests should be described solely by name; describe more complex techniques in the Methods section.*
☒	☐	A description of all covariates tested
☒	☐	A description of any assumptions or corrections, such as tests of normality and adjustment for multiple comparisons
☐	☒	A full description of the statistical parameters including central tendency (e.g. means) or other basic estimates (e.g. regression coefficient) AND variation (e.g. standard deviation) or associated estimates of uncertainty (e.g. confidence intervals)
☒	☐	For null hypothesis testing, the test statistic (e.g. *F*, *t*, *r*) with confidence intervals, effect sizes, degrees of freedom and *P* value noted<br>*Give P values as exact values whenever suitable.*
☒	☐	For Bayesian analysis, information on the choice of priors and Markov chain Monte Carlo settings
☒	☐	For hierarchical and complex designs, identification of the appropriate level for tests and full reporting of outcomes
☒	☐	Estimates of effect sizes (e.g. Cohen's *d*, Pearson's *r*), indicating how they were calculated

Our web collection on statistics for biologists contains articles on many of the points above.

Software and code

Policy information about availability of computer code

Data collection	Software used for data collection includes the commercially available Nikon NIS Elements AR imaging software.
Data analysis	Software used for data analysis includes the open source image analysis software ImageJ v 2.1.0/1.53c; NCBI BLAST suites; Bowtie for Illumina v 1.1.2

For manuscripts utilizing custom algorithms or software that are central to the research but not yet described in published literature, software must be made available to editors and reviewers. We strongly encourage code deposition in a community repository (e.g. GitHub). See the Nature Research guidelines for submitting code & software for further information.

Data

Policy information about availability of data

All manuscripts must include a data availability statement. This statement should provide the following information, where applicable:

- Accession codes, unique identifiers, or web links for publicly available datasets
- A list of figures that have associated raw data
- A description of any restrictions on data availability

The raw sequencing data for the Tn-seq experiments has been uploaded to the Sequence Read Archive (https://www.ncbi.nlm.nih.gov/sra): PRJNA716813. Additional data that support the findings of this study are available in the Source Data file.

Field-specific reporting

Please select the one below that is the best fit for your research. If you are not sure, read the appropriate sections before making your selection.

☒ Life sciences ☐ Behavioural & social sciences ☐ Ecological, evolutionary & environmental sciences

For a reference copy of the document with all sections, see nature.com/documents/nr-reporting-summary-flat.pdf

Life sciences study design

All studies must disclose on these points even when the disclosure is negative.

Sample size	Sample sizes were chosen based on historical data and no statistical methods were used to predetermine sample size (Ellison et al. Nature Microbiology 2018).
Data exclusions	No data were excluded from the analysis
Replication	All data are from a minimum of two independent experiments. All replication attempts were successful.
Randomization	This is not relevant to our study since our samples are all from individual cultures.
Blinding	Blinding is not relevant to our study since our samples are all from individual cultures.

Reporting for specific materials, systems and methods

We require information from authors about some types of materials, experimental systems and methods used in many studies. Here, indicate whether each material, system or method listed is relevant to your study. If you are not sure if a list item applies to your research, read the appropriate section before selecting a response.

Materials & experimental systems

n/a	Involved in the study	
☐	☒	Antibodies
☒	☐	Eukaryotic cell lines
☒	☐	Palaeontology and archaeology
☒	☐	Animals and other organisms
☒	☐	Human research participants
☒	☐	Clinical data
☒	☐	Dual use research of concern

Methods

n/a	Involved in the study	
☒	☐	ChIP-seq
☒	☐	Flow cytometry
☒	☐	MRI-based neuroimaging

Antibodies

Antibodies used	mouse monoclonal α-FLAG antibody (Sigma Aldrich cat#F1804) or mouse monoclonal α-GFP antibody (Roche) mouse monoclonal α-RpoA (BioLegend cat#663104) primary antibodies; IRDye-680RD goat α-mouse secondary antibody (Licor cat#925-68070)
Validation	The α-FLAG and α-GFP antibodies were validated by comparing tagged strains to the parent strain lacking a genetically encoded tag. For example, in Figure 3d, the parent strain lacking genetically encoded tags has no band present where FLAG-tagged strains do. In Figure 4d, the FLAG-pilB control strain that lacks a GFP tag does not have signal at the size of the GFP-tagged protein. RpoA antibodies were validated by comparing bands in blots treated with or without α-RpoA antibody (see Figure 3d treated with antibody compared to Figure 4d not treated with antibody in Source Data file).
